# New constraints on the postglacial shallow-water carbonate accumulation in the Great Barrier Reef

**DOI:** 10.1038/s41598-021-04586-w

**Published:** 2022-01-18

**Authors:** Gustavo Hinestrosa, Jody M. Webster, Robin J. Beaman

**Affiliations:** 1 Geocoastal Research Group, School of Geosciences, The University of Sydney, Sydney, Australia; 2 College of Science and Engineering, James Cook University, Cairns, Australia

**Keywords:** Biogeochemistry, Climate change, Climate sciences, Palaeoceanography, Geomorphology, Sedimentology, Carbon cycle, Biogeochemistry, Element cycles

## Abstract

More accurate global volumetric estimations of shallow-water reef deposits are needed to better inform climate and carbon cycle models. Using recently acquired datasets and International Ocean Discovery Program (IODP) Expedition 325 cores, we calculated shallow-water CaCO_3_ volumetrics and mass for the Great Barrier Reef region and extrapolated these results globally. In our estimates, we include deposits that have been neglected in global carbonate budgets: Holocene *Halimeda* bioherms located on the shelf, and postglacial pre-Holocene (now) drowned coral reefs located on the shelf edge. Our results show that in the Great Barrier Reef alone, these drowned reef deposits represent ca. 135 Gt CaCO_3_, comparatively representing 16–20% of the younger Holocene reef deposits. Globally, under plausible assumptions, we estimate the presence of ca. 8100 Gt CaCO_3_ of Holocene reef deposits, ca. 1500 Gt CaCO_3_ of drowned reef deposits and ca. 590 Gt CaCO_3_ of *Halimeda* shelf bioherms. Significantly, we found that in our scenarios the periods of pronounced reefal mass accumulation broadly encompass the occurrence of the Younger Dryas and periods of CO_2_ surge (14.9–14.4 ka, 13.0–11.5 ka) observed in Antarctic ice cores. Our estimations are consistent with reef accretion episodes inferred from previous global carbon cycle models and with the chronology from reef cores from the shelf edge of the Great Barrier Reef.

## Introduction

The role of calcium carbonate deposits in the carbon cycle, and the influence on climate change during the late-Quaternary is poorly constrained. The fifth assessment report of the Intergovernmental Panel on Climate Change^1^ identifies the major contributors for atmospheric CO_2_ (atm-CO_2_) concentration changes from the last glacial maximum (LGM) to present. The authors assigned a *medium* degree of confidence to the current estimates of atm-CO_2_ contributions from coral reef accretion and carbonate compensation depth changes. This uncertainty derives not just from the use of proxy data and their limited availability, but from the complex relationships between the carbon and other biogeochemical cycles. Such uncertainty is reflected in the range of the contributions to postglacial atm-CO_2_ attributed to shallow-water reefs in global carbon models (− 9 to 30 ppm^2–5^).

The role that coral reefs may have played in this process has been termed the *coral reef hypothesis *^5–7^. This hypothesis proposes that the increase of the atm-CO_2_ is at least partly due to the enhanced shallow-water CaCO_3_ accretion by corals. This hypothesis relies on the availability of new areas of marine flooded shelf during the last transgression and on the consequent increase in coral reef development. This would have changed the alkalinity balance, at least locally, and ultimately increasing the transfer of CO_2_ to the atmosphere^6,8^. Because of the reduced marine shelf area during glacial times and the subsequent increase in reef area from glacial to Holocene times, it is assumed that the coral reefs acted as secondary amplifiers—not precursors—of a climatic change that had already initiated^9,10^ with early atm-CO_2_ rise generally preceding global surface temperature increase^11^.

The *coral reef hypothesis* is supported by the extensive coral reefs of Holocene age worldwide^12–16^. Moreover, a possible lower contribution from terrestrial sources in this same period^3,17^ argues in favour of alternative carbon sources, such as that represented by reef accretion. Simplified box models^6,7^ have suggested that the activity of the coral reefs can explain a significant rise of the atm-CO_2_ during postglacial times. However, the dissolution ratios, accretion rates and calcite saturation depth informing these models are poorly constrained and they possibly overestimate the total carbon derived from corals.

More complex models have considered the effect of coral reefs in postglacial atm-CO_2_^2–5,18,19^. Notably, Ridgwell et al.^5^ inferred two possible minor episodes of global reef growth from 17.0 to 13.8 ka BP and from 12.3 to 11.2 ka BP. With no conclusive evidence available at that time for such global reef growth episodes^20^, they attributed the increase in CO_2_ to changes in the biogeochemical properties of the Southern Ocean surface.

Interestingly, recent evidence in Tahiti and the Great Barrier Reef (GBR) has revealed glacial and early-postglacial (30 to 10 ka BP) coral reef episodes in line with those inferred intervals (IODP Expeditions 310 & 325^21,22^). Globally, drowned reefs may constitute an important fraction of postglacial carbonate^21,23,24^ and an alternative earlier source of postglacial carbon. *Halimeda* bioherms are another contributing component, with recent investigations from the GBR suggesting they are volumetrically relevant in postglacial carbonate budgets^25–27^.

Estimations of global and regional reef carbonate area and volume have been attempted using a range of assumptions^13,15,28^ and applying parameters (e.g. reef area) with large associated uncertainties. New datasets are, however, providing valuable constraints to these parameters within the GBR region. For example, new GIS datasets of reef boundaries^29^ have the potential to improve the estimations of reef area. Additionally, the recent surveys and investigations on the drowned shelf edge reefs of the GBR represent the most complete dataset investigating drowned reefs: IODP Exp. 325 well-dated fossil reef cores^30,31^, seismic lines^32,33^, multibeam bathymetry^34^, and surface sediment and rock dredge samples^35,36^. New detailed bathymetry and interpretations are also providing new constraints on the spatial distribution and volume of *Halimeda* bioherms in the northern GBR^26,27^.

In this paper we investigate the impact of drowned coral reefs and *Halimeda* deposits on regional (GBR shelf, northeastern Australia) and global postglacial shallow-water CaCO_3_ budgets. Our scientific objectives are to: (1) estimate the volume, mass and timing of postglacial shallow calcium carbonate deposition across the entire GBR using the most recent GIS datasets, data from two IODP Exp. 325 control zones from the shelf-edge reef system and the regional volume of the *Halimeda* bioherms; (2) extend the resulting volumetric and mass estimates globally based on assumptions ground-truthed in the GBR; and (3) compare our results with past regional and global volumetric and mass estimates.

## Regional setting

**Figure 1:**
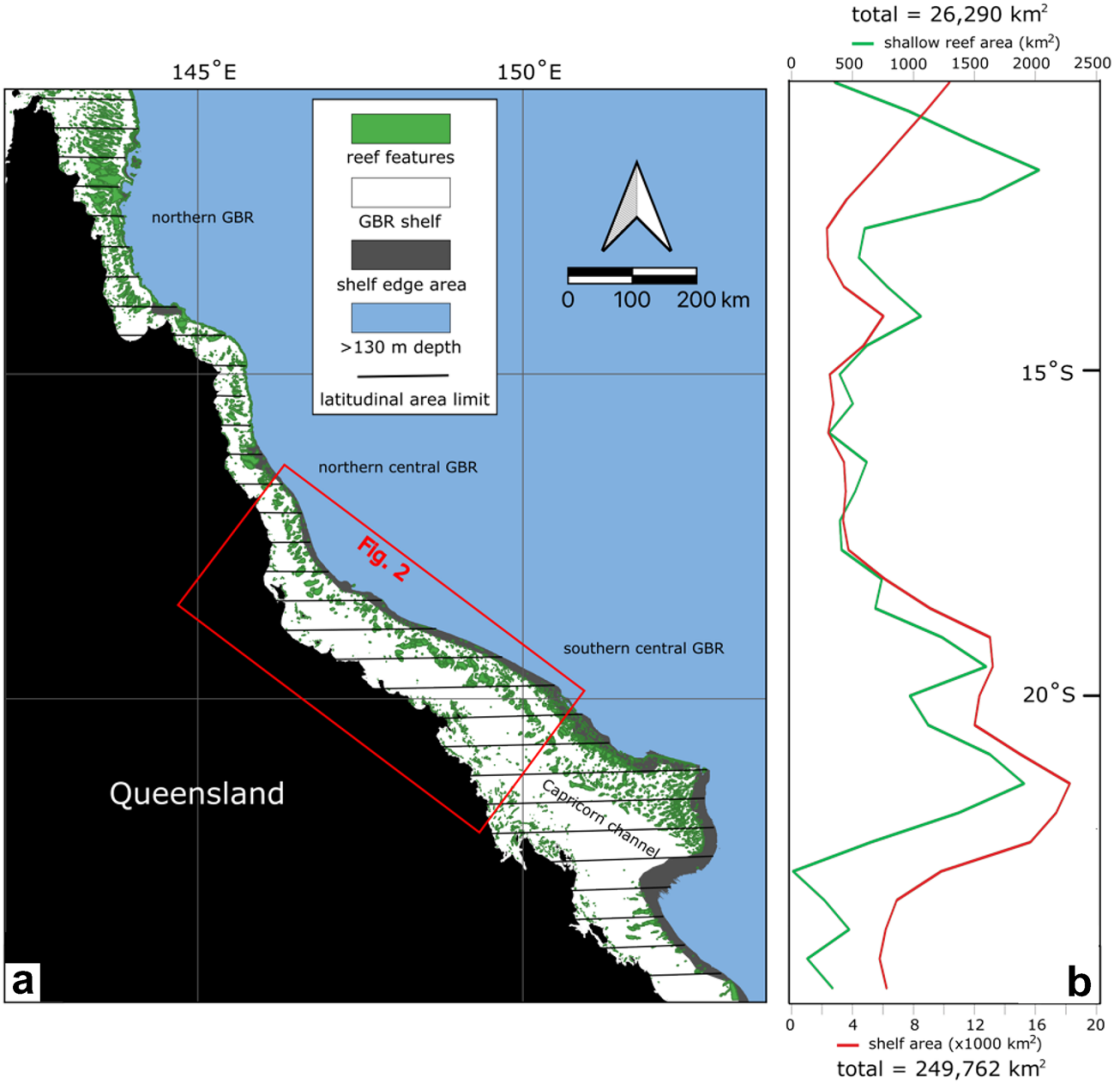
Regional location: (**a**) Present day coastline and bathymetry of the GBR shelf, from Fraser Island in the south to Cape York in the north. Note the shallow reef presence map as interpreted from satellite imagery and bathymetric data^29,50^ and the latitudinal areas. (**b**) Shelf area and Holocene reef area as calculated for each latitudinal zone. Note the high correlation of the two curves in the central GBR.

The GBR shelf along the northeastern coast of Australia (Fig. 1) accommodates a thick succession of reef deposits over the last 600 ± 280 ky^37,38^ controlled by major glacial-interglacial sea-level fluctuations. The last glacial-interglacial fluctuation initiated during the LGM^21,30^ when sea level was approaching minimum levels (120–130 m below present)^31,39^.

**Figure 2:**
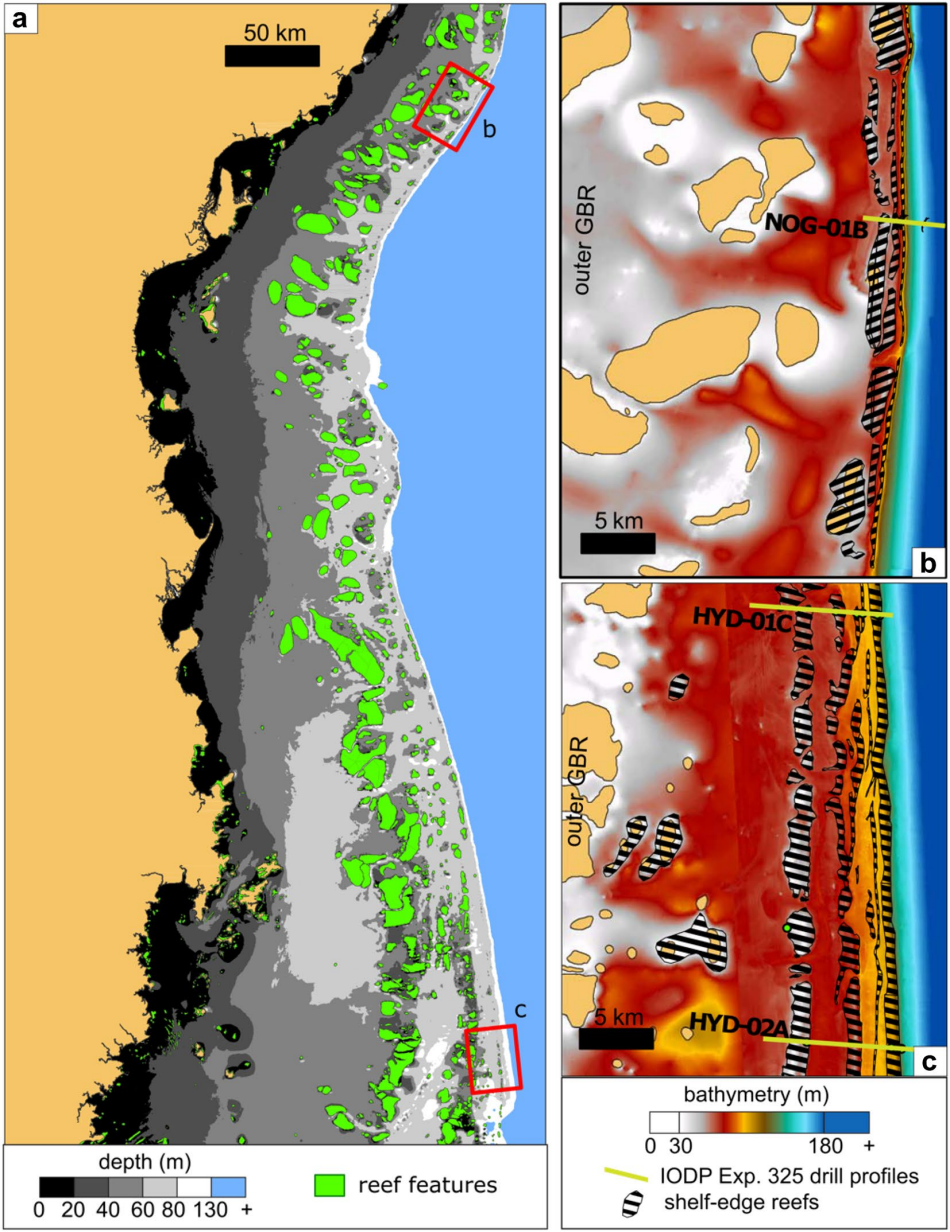
Central GBR and control zones: (**a**) Bathymetry of the central GBR with shallow reef presence map as interpreted from satellite imagery and bathymetric data^29^, and the locations of the control zones in the vicinity of Noggin Passage and Hydrographers Passage. (**b**) Noggin Passage in the northern central GBR has a narrower shelf edge reef area. (**c**) Hydrographer’s Passage in the southern central GBR. Note the reef area as estimated for the shelf edge and the location of the seismic and IODP Exp. 325 drilling transects NOG-01B, HYD-01C, HYD-02A^30,40^.

As the glaciation ended and sea level rose during the postglacial, extensive fringing- and barrier-reef structures developed along the shelf edge of the GBR until ca. 10 ka BP^21,40^. These reefs currently lie between 40 and 130 m below present sea level and extend for more than 2000 km from the northern to the southern central GBR and possibly farther^34,41,42^ (Fig. 2). Beyond the 130 m depth contour, the shelf edge fore-reef sediments give way to hemipelagic sediments of carbonate and terrigenous origin on the upper continental slope^43–45^. Evidence suggests that the inter-reef areas of the shelf edge are covered by a relatively thin (0–5 m) layer of carbonate sands and mud, dominated by *Halimeda* fragments, foraminifera, mollusks and bryozoans^35,36,46,47^.

The shallower structures of the modern GBR were colonized after ca. 9 ka BP following the demise of the shelf-edge reefs. Since then, extensive reef development has occurred along the whole GBR from the vicinity of Fraser Island in the south, to Cape York in the north^20,48^. Both Pleistocene and Holocene reef deposits in the GBR display local and regional variations that are the expression of broader physiographic trends and of the physical processes linked to the postglacial marine flooding^34,40,49^.

## Results and discussion

### Holocene carbonate deposits

We estimated the areal trends of the Holocene reefs in the GBR. Reef area was estimated from GIS layers containing polygons representing the outline of the Holocene reefs (Figs. 1 and 2). This layer was obtained from a detailed interpretation of recently available satellite images and shallow bathymetry^29,50^, and excluding continental islands and shelf-edge reefs. These features were sliced into latitudinal 50 km wide slices to assess latitudinal variations. We assumed that the reef area polygons represented the main reef and bioclastic deposits directly related to Holocene reef growth. However, unaccounted fore- and back-reef aprons may constitute a significant portion of the reefal carbonate volume^15,51^.

**Figure 3:**
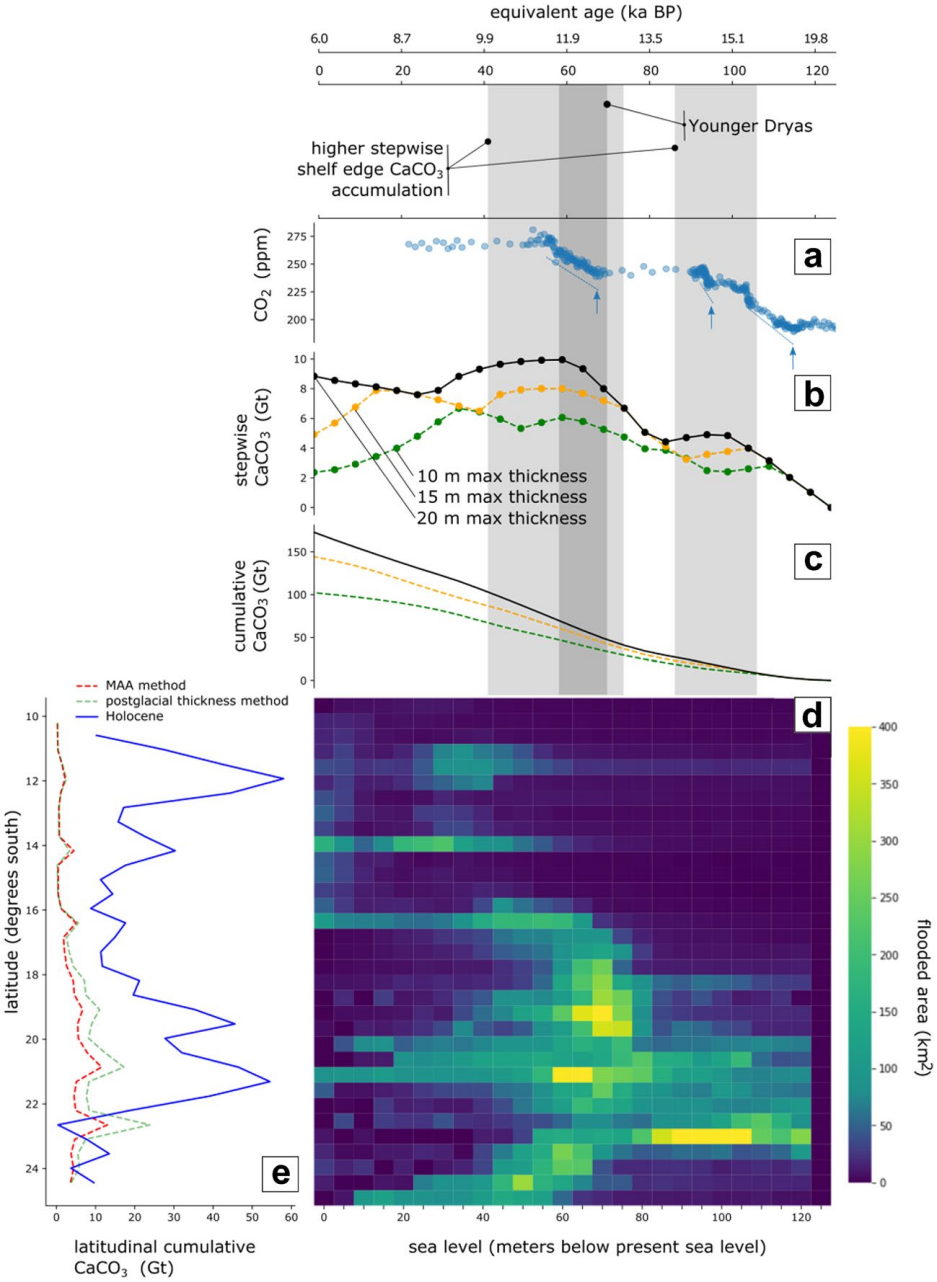
Summary of shelf edge reef deposits in the GBR: (**a**) atm-CO_2_^67^ data from Antarctic ice cores, intervals of increased rate are highlighted in blue; (**b**) stepwise shelf edge reef deposits; (**c**) cumulative shelf edge CaCO_3_ increase for different maximum accretion thicknesses, (**d**) shelf edge CaCO_3_ mass deposits for every latitudinal zone and for every past sea level 5 m increase; (**e**) comparison of the latitudinal distribution of CaCO_3_ mass deposits (best-estimate case) for the Holocene reefs and the shelf edge reefs according to the two applied methods. Notice the increase of the values between 18° and 22° S where the shelf is wider.

We estimated the mass of the Holocene CaCO_3_ deposits by multiplying the area derived from the interpreted GIS layer by the thickness derived from historical reef cores that have drilled through the Holocene (Appendix 1) and petrophysical parameters (Aragonite density, ρ_A_ = 2930 kg m^−3^ and formation porosity, Φ_R_ = 35%^30^). The volumetric and mass calculations were also performed at a sub-regional level in 50 km wide latitudinal slices that allowed reconstruction of latitudinal trends (Fig. 3e, Appendix 2).

Our area estimations show that Holocene reefs occupy approximately 10% of the total GBR shelf area, following a latitudinal trend that correlates with the total shelf area, especially in the central GBR (R^2^ = 0.84) between 14° and 21° S (Figs. 1, 3e). This reflects the direct relationship between substrate availability on the shelf and carbonate accumulation. Interestingly, in the northern GBR (near 12° S) the proportion of Holocene reef area-to-shelf area increases due to the wide (up to 30 km) reef structures. The seemingly obvious relationship is, however, a complex one and highly dependent on environmental variables (terrigenous flux, circulation, antecedent substrate, etc.) that can determine the spatial distribution of the carbonate deposits^40,49,52–54^.

Our new GBR Holocene CaCO_3_ mass estimates (Table 1) fall between the figures by Rees^13^ and Kinsey and Hopley^55^. The Rees^13^ reef area estimate of 44,920 km^2^, based on Spalding et al.^56^ dataset, is larger than the area presented in this study (26,290 km^2^, Table 2). Their Holocene CaCO_3_ estimate is consequently higher (1709 Gt CaCO_3_) than our best estimate of 751 Gt CaCO_3_. However, Rees^13^ include reefs in the Australian region that are not part of the GBR system sensu stricto. The Kinsey and Hopley^55^ estimated reef area value is more in agreement with our figure: ca. 20,000 km^2^. A more recent estimate places the GBR’s shallow-water reef area at 16,110 km^2^^50^, but they do not include sediment wedges associated with these reefs, which are found at greater depths and constitute up to the double of the reef framework volume^15,51^. If we calculate the mass areal accumulation (MAA) using Rees^13^ estimates (Table 1) the resulting MAA is much higher (38,045 kg m^−2^) than even the highest MAA in Hydrographers Passage (27,648 kg m^−2^, Table 2), which seems unlikely. Even applying matching reef areas to Rees^13^ and Kinsey and Hopley^55^ results, these past estimates probably overestimate the Holocene carbonate deposits in the GBR.

**Table 1:** GBR estimates of CaCO_3_ volume, mass and MAA for the Holocene, shelf edge reefs and *Halimeda* bioherms of the GBR.

Study	Kinsey and Hopley^55^	Rees^13^	This study								
Feature	Holocene reefs	Holocene reefs	Holocene reefs			SERs (postglacial thickness method)					
Dataset/observations	CaCO_3_ mass based on accumulation rate (0.049 Gt CaCO_3_ y^−1^)	Rees^13^	Interpretation of reef features, Harris et al.^50^			Beaman^57^, Webster et al.^30^, Hinestrosa et al.^40,47^ and Webster et al.^21^					
Scenario	8 to 0 ka BP	10 to 0 ka BP	Min	Best est	Max	Min	Best Est	Max	100% reef-area ratio		
Reef area (km^2^)	**20,055**	**44,920**	**26,290**			**2870**	**5741**	**11,482**	**28,704**		
Average reef thickness (m)	13	26	**10**	**15**	**20**	**10**	**15**	**20**	**15**		
Total volume above 130 mbsl (km^3^)	270	1179	273	394	526	27	78	192	391		
Porosity (%)	**50**	**50**	**35**	**35**	**35**	**35**	**35**	**35**	**35**		
Net CaCO_3_ volume (km^3^)	135	589	177	256	342	18	51	125	254		
Density (kg m^−3^)	**2900**	**2900**	**2930**	**2930**	**2930**	**2930**	**2930**	**2930**	**2930**		
Net CaCO_3_ mass (Gt CaCO_3_)	**392**	**1709**	520	751	1001	52	149	366	745		
Mass areal accumulation (kg m^−2^)	19,546	38,045	19,776	28,568	38,090	18,047	25,943	31,914	25,943		
Reef-cover	9%	–	10.5%			**10%**	**20%**	**40%**	**100%**		

Study	This study										
Feature	SERs (MAA method)				Halimeda mounds	Total postglacial (reef)			Total postglacial		
Dataset/observations	Beaman^57^, Webster et al.^30^ and Hinestrosa et al.^40,47^				McNeil et al.^26^	Holocene values + average of SERs values			Holocene values + average of SERs values + *Halimeda* preliminary estimate		
Scenario	Min	Best est	Max	100% reef-area ratio	Only N and central GBR	Min	Best est	Max	Min	Best est	Max
Reef area (km^2^)	**2870**	**5741**	**11,482**	**28,704**	**6111**	**29,161**	**32,031**	**37,772**	**35,272**	**38,142**	**43,883**
Average reef thickness (m)	8	11	15	11	**8.4**	14–25	14–25	14–25	5–20	5–25	5–25
Total volume above 130 mbsl (km^3^)	22	61	167	305	**51.5**	298	464	705	349	515	757
Porosity (%)	**35**	**35**	**35**	**35**	**58**	35	35	35	35–50	35–50	35–50
Net CaCO_3_ volume (km^3^)	14	40	108	198	**20**	193	302	458	213	321	478
Density (kg m^−3^)	**2930**	**2930**	**2930**	**2930**	**2930**	2930	2930	2930	2930	2930	2930
Net CaCO_3_ mass (Gt CaCO_3_)	42	116	317	581	55	567	884	1343	622	939	1398
Mass areal accumulation (kg m^−2^)	**14,509**	**20,245**	**27,648**	**20,245**	9000	19,432	27,586	35,564	17,625	24,609	31,865
Reef-cover	**10%**	**20%**	**40%**	**100%**	–	10%*	20%*	40%*	10%*	20%*	40%*

Rees^13^ and Kinsey and Hopley^55^ estimates are shown for comparison. Bold highlight the parameters applied for calculation.

*Values used for SERs only.

**Table 2:** Deposits at the control zones of Hydrographer’s and Noggin Passages: total area, CaCO_3_ volume, CaCO_3_ mass and mass areal accumulation for different geomorphic zones^47^.

Study area		**Hydrographers Passage**								
Stratigraphic unit		Unit 1 (postglacial)								
Geomorphic zone		Entire study area			Inner barrier			Outer barrier		
Velocity scenario		Min	Best est	Max	Min	Best est	Max	Min	Best est	Max
Area (km^2^)	Polygons digitized on geomorphic maps	422	422	422	42	42	42	29	29	29
Total volume (km^3^)	Calculations above 130 mbsl	2.71	3.18	3.64	0.41	0.57	0.74	0.31	0.42	0.53
Net carbonate volume (km^3^)	Porosity = 35%	1.76	2.07	2.36	0.27	0.37	0.48	0.20	0.27	0.35
Net carbonate mass (Gt CaCO_3_)	Density = 2930 kg m^−3^	5.16	6.06	6.93	0.78	1.09	1.40	0.59	0.80	1.02
Mass areal accumulation (kg m^−2^)	Area as indicated	12,235	14,370	16,414	18,682	26,028	33,419	20,393	27,648	35,003
Reef area cover		18.8%								

Study area		**Hydrographers Passage**								
Stratigraphic unit		Unit 1 (postglacial)								
Geomorphic zone		Inner + outer platform			Terrace + shelf-break			All barrier (inner + outer)		
Velocity scenario		Min	Best est	Max	Min	Best est	Max	Min	Best est	Max
Area (km^2^)	Polygons digitized on geomorphic maps	240	240	240	111	111	111	71	71	71
Total volume (km^3^)	calculations above 130 mbsl	0.98	1.02	1.06	1.01	1.17	1.31	0.72	1.00	1.27
Net carbonate volume (km^3^)	Porosity = 35%	0.64	0.66	0.69	0.66	0.76	0.85	0.47	0.65	0.83
Net carbonate mass (Gt CaCO_3_)	Density = 2930 kg m^−^3	1.86	1.94	2.02	1.92	2.23	2.49	1.38	1.89	2.42
Mass areal accumulation (kg m^−2^)	Area as indicated	7767	8092	8406	17,325	20,062	22,438	19,381	26,690	34,066
Reef area cover		18.8%								

Study area		**Noggin Passage**								
Stratigraphic unit		Unit 1 (postglacial)								
Geomorphic zone		Entire study area			Inner barrier			Outer barrier		
Velocity scenario		Min	Best est	Max	Min	Best est	Max	Min	Best est	Max
Area (km^2^)	Polygons digitized on geomorphic maps	85	85	85	10	10	10	18	18	18
Total volume (km^3^)	Calculations above 130 mbsl	0.49	0.54	0.58	0.07	0.08	0.10	0.14	0.16	0.18
Net carbonate volume (km^3^)	Porosity = 35%	0.32	0.35	0.38	0.05	0.06	0.06	0.09	0.10	0.12
Net carbonate mass (Gt CaCO_3_)	Density = 2930 kg m^−3^	0.93	1.02	1.11	0.13	0.16	0.19	0.26	0.31	0.35
Mass areal accumulation (kg m^−2^)	Area as indicated	10,956	11,987	13,086	13,222	16,087	18,972	14,726	17,134	19,586
Reef area cover		17.8%								
Average mass areal accumulation [kg m^−2^]	Barriers and shelf break	20,245								

Study area		**Noggin Passage**								
Stratigraphic unit		Unit 1 (postglacial)								
Geomorphic zone		Inner + outer platform			Terrace + shelf-break			All barrier (inner + outer)		
Velocity scenario		Min	Best est	Max	Min	Best est	Max	Min	Best est	Max
Area (km^2^)	Polygons digitized on geomorphic maps	26	26	26	31	31	31	28	28	28
Total volume (km^3^)	Calculations above 130 mbsl	0.05	0.06	0.06	0.23	0.23	0.24	0.21	0.25	0.28
Net carbonate volume (km^3^)	Porosity = 35%	0.03	0.04	0.04	0.15	0.15	0.16	0.14	0.16	0.18
Net carbonate mass (Gt CaCO_3_)	Density = 2930 kg m^−3^	0.10	0.11	0.11	0.44	0.45	0.46	0.40	0.47	0.54
Mass areal accumulation (kg m^−2^)	Area as indicated	3817	3996	4342	14,146	14,509	14,886	14,186	16,758	19,365
Reef area cover		17.8%								
Average mass areal accumulation [kg m^−2^]	Barriers and shelf break	20,245								

*Reef area ratio* in the area comprised between the outer GBR fore-reef and the 130-m contour is similar in both sites. These figures are based on seismic three-dimensional reconstructions, geomorphic interpretations and core data^30,34,40,47^.

### Shelf edge reef CaCO_3_ accumulation

The volume and mass of the shelf edge deposits at a regional scale were ground-truthed in two control zones along the shelf edge of the GBR: Hydrographers Passage and Noggin Passage (Fig. 2). Here, extensive IODP drilling, bathymetric and seismic surveys^30,34,40,47^ provided valuable data for the calibration of the parameters required for the regional reconstruction of volumetrics and mass: *reef area ratio*, *formation volume* from seismic imaging, *mass areal accumulation* (*MAA*), *vertical accretion rate*, the *maximum cumulative thickness* and petrophysical parameters. A regional bathymetric dataset^57^ provided the basis for the reconstruction for the postglacial marine *flooded area* in the GBR (analogous to Hinestrosa et al.^49^).

**Figure 4:**
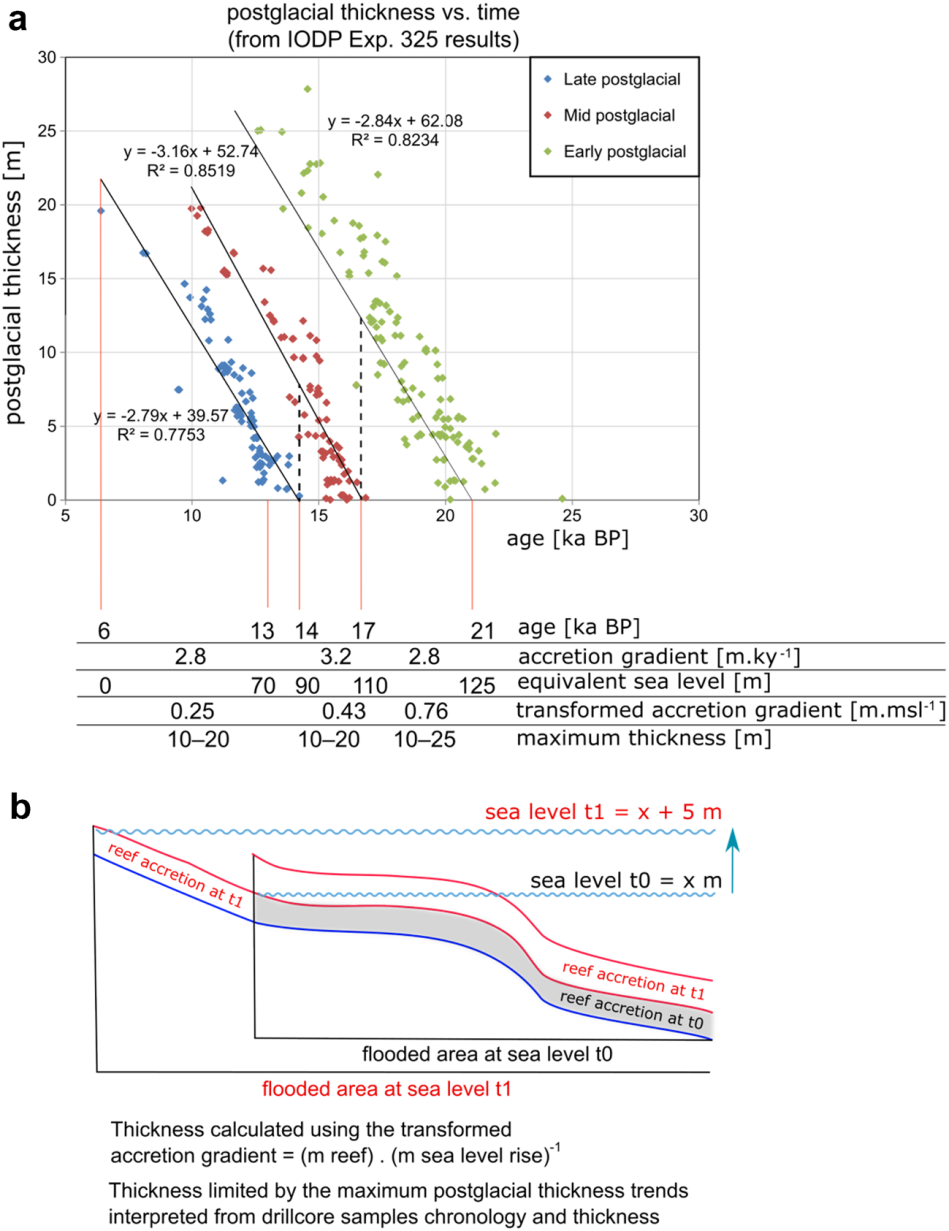
Description of the postglacial thickness method for the calculation of reef deposits: (**a**) Vertical accretion rates (VA) and maximum accretion thickness were extracted from trends of postglacial thickness vs. geological age using the dated core samples from the IODP Exp. 325 (Webster et al.^21^, Appendix 3). These rates were converted into an equivalent rate relative to the past sea level increase (transformed accretion rate VA_SL_) using a composite sea level curve (Fig. 5, Appendix 6). (**b**) The marine-flooded areas for each postglacial sea level (FA_SL_)^49^ were multiplied by the thickness corresponding to one sea level step, according to the previously calculated rate VA_SL_. Flooded areas were not allowed to accumulate reef thickness beyond the maximum observed in (**a**). This can be represented as a thickness matrix (**b**) where each sea level step (t0, t1, …, tn) has a thickness vector applicable to the different flooded-area polygons (see Appendix 5 for full calculations).

**Figure 5:**
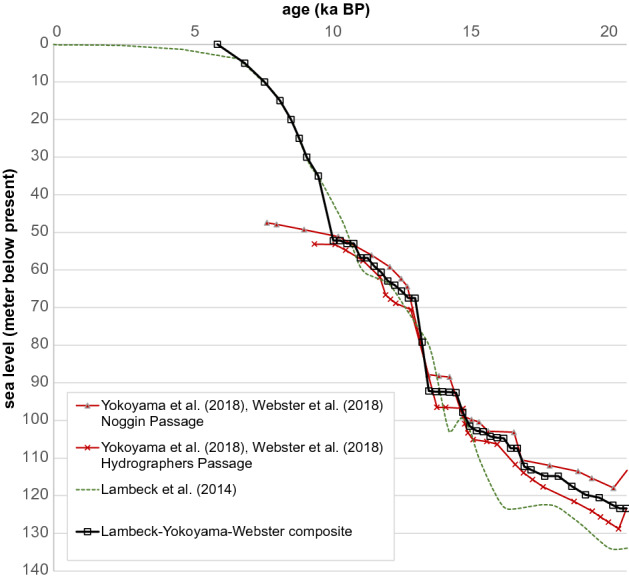
Composite sea level curve: constructed based on data in Lambeck et al.^39^ and Webster et al.^21^, Yokoyama et al.^31^. This sea-level curve was used to translate between past sea levels and geological time, allowing a possible temporal reconstruction of the CaCO_3_ deposition trends in the shelf edge of the GBR.

Two methods were applied to obtain the volumetrics and mass of the shelf-edge deposits: (1) the mass areal accumulation method and (2) the postglacial thickness method. The former is a bulk calculation of volume based on *flooded area* and the accumulation represented by the *MAA*, scaled back by the proportion of bathymetric surface covered by reefs (*reef area ratio*) without considering any temporal evolution*.* The latter, the postglacial-thickness method*,* attempts to reconstruct the temporal evolution of the reef accretion by considering the change in *flooded area* since the LGM. It is layer-based, with each layer corresponding to a 5 m sea-level step in which data-derived thickness constraints (Fig. 4a, Appendix 3) are applied in such a way that vertical reef accumulation does not exceed observed thicknesses (Fig. 4b). It relies on the assumption that the *reef area ratio*, *vertical accretion rate* and *maximum cumulative thickness* values observed in the control zones can be extended to other locations along the GBR shelf edge. A composite sea-level curve based on Lambeck et al.^39^ and Yokoyama et al.^31^ (Fig. 5, Appendix 6) enabled the translation between past sea levels and geological ages to reconstruct the temporal evolution of the deposits.

The estimates obtained applying the mass areal accumulation method are lower than those estimated by applying the postglacial thickness method but are within the same order of magnitude. Not surprisingly, the application of both methods results in similar latitudinal trends (Fig. 3e) because in both the flooded area is a direct factor in the calculations.

#### Control zones

In the control zones, Noggin Passage and Hydrographers Passage (Fig. 2), ca. 20% of the shelf edge is covered in reef structures (i.e., *reef area ratio* = ca. 20%). This proportion is almost twice the *reef area ratio* estimated at a regional scale for the shallower, Holocene reefs when considering the whole of the GBR shelf.

In both control zones (Table 2) we found that reef areas have MAA values above 10,000 kg m^−2^, whilst inter-reef areas display MAA values that are an order of magnitude lower (7767–8406 kg m^−2^). The southern control zone (Hydrographers Passage) has a higher average MAA (e.g., 20,393–35,003 kg m^−2^ in the outer barrier) than the northern site (Noggin Passage) in all the geomorphic areas assessed (e.g., 14,726–19,586 kg m^−2^ in the outer barrier). This is consistent with a thinner reef veneer (< 10 m) in the terrace and outer geomorphic areas and less distinguishable barrier structures found at Noggin Passage^40^.

#### Shelf edge trends

Volumetrically, the carbonate deposits of the shelf edge wane in the northern GBR when compared to the central and southern GBR (Fig. 3d, e). In the northern GBR, the Holocene and the shelf edge pre-Holocene reef trends are also contrasting: the Holocene reef deposits increase dramatically near 12° S, whereas the shelf edge deposits at those latitudes diminish, possibly due to limited substrate availability^40^. On the contrary, in the southern-central GBR (e.g. Hydrographers Passage control zone) wider and a more gentle gradient provides more substrate availability^34^.

It is useful to establish some comparisons to appreciate the magnitude of the shelf edge deposits. At the shelf edge, our estimates suggest that the area covered by reef formations is between 3000 and 11,000 km^2^ (Table 1), which would represent 8% (minimum case) to 30% (maximum case) of the total area occupied by all the banks in Harris et al.^50^, and between 12% (minimum case) and 48% (maximum case) of the area occupied by banks with no Holocene cover in that same study. Kleypas^58^ inferred that the global area available for reef growth during the LGM lowstand was approximately 20% of that available today. In the GBR, that figure is at least within the same order of magnitude of our estimations: a maximum shelf edge flooded area of ca. 29,523 km^2^ (best estimate, Table 1) representing 12% of the whole GBR shelf (ca. 249,762 km^2^).

### Other carbonate deposits in the GBR

*Halimeda* bioherms are a significant component of the region’s postglacial carbonate budget. A comparison of the most up-to-date morphometric data^27^ shows that the postglacial *Halimeda* deposits are equivalent in mass to 5.5–10.5% of the Holocene reef mass on the GBR shelf (Table 1). *Halimeda* can form mounds in inter-reef areas of the GBR up to a thickness of 20 m^25–27,59,60^. Recent reviews of published and new high-resolution bathymetry have revealed that at least 6000 km^2^ of the northern and central GBR are covered by *Halimeda* bioherms^26,27^. This is a considerable increase from the ca. 2000 km^2^ from previous estimations^59^.

*Halimeda*-like morphologies have also been detected on the shelf edge in seismic profiles^47^ and *Halimeda* floatstones recovered in dredges from the shelf edge dated to 11.8–7.2 ka (D24B, D22, D11B in Abbey et al.^36^). Despite these new constraints, questions remain about the extent of pre-Holocene *Halimeda* deposits, particularly at the shelf edge.

### Global and regional estimates

We extrapolated the estimates and trends of CaCO_3_ deposits for the GBR to the entire globe using the reef area estimate of Spalding et al.^56^. Using this area, we applied the parameters ground-truthed in the GBR dataset. The *global reef area* (RA_GLOBAL_) was multiplied by average thickness and petrophysical parameters (ρ_A_, Φ_R_) to obtain global postglacial CaCO_3_ deposits. We accounted for the drowned postglacial reefs by applying two factors to the global Holocene estimates: a factor based in the ratio of shelf edge-to-Holocene reef area (area adjustment factor, AF_A_) and a factor based in the mass ratio (mass adjustment factor, AF_M_) (Table 3). These factors and the global extrapolation as such, have large associated uncertainties given the gaps in knowledge of total global extent, accretion trends and morphology of the less accessible postglacial drowned reefs. We also calculated the global volumetrics and mass globally using the reef areas from past studies (Tables 4, 5; Appendix 4).

**Table 3:** Global estimates of shallow-water reefs, shelf edge reefs and *Halimeda* bioherms as extrapolated from the proportions of the different features relative to the Holocene reefs of the GBR.

Study	This study				
Feature	Global Holocene reefs	Global shelf-edge reefs (from GBR mass ratio)	Global shelf-edge reefs (from GBR area ratio)	Global *Halimeda* mounds (from GBR mass ratio)	Total postglacial CaCO_3_
Dataset/observations/parameters	Area in Spalding et al.^56^, other parameters from this study	This study, shelf edge/Holocene mass adjustment factor = 18%	This study, shelf edge/Holocene area adjustment factor = 22%	McNeil et al.^27^	Global Holocene + shelf-edge + Halimeda mounds
Timing (ka BP)	8 to 0	19 to 10	19 to 10	8 to 0	19 to 0
Reef area (km^2^)	**284,000**	51,120	**62,480**	57,295	398,095
Average reef thickness (m)	**15**	**15**	**15**	**8**	5–15
Total volume > 130 mbsl (km^3^)	4260	767	937	481	5593
Porosity (%)	**35**	**35**	**35**	**58**	35–50
Net CaCO_3_ volume (km^3^)	2769	498	609	202	3525
Density (kg m^−3^)	**2930**	**2930**	**2930**	**2930**	2930
Net CaCO_3_ mass (Gt CaCO_3_)	8113	**1460**	1785	**592**	10,328
Net CaCO_3_ accumulation rate (Gt CaCO_3_ y^−1^)	1.0	0.2	0.2	0.1	0.6
Mass areal accumulation (kg m^−2^)	28,568	28,568	28,568	10,337	25,944

Bold highlight the parameters applied in each case from which the rest of the values were derived.

**Table 4:** Extrapolations of global CaCO_3_ deposits applying the parameters of this study (Table 3) but using the broad range of past global reef area estimates shown in Table 5.

Calculation	Total postglacial reefal (and Halimeda) CaCO_3_, including pre-Holocene drowned reefs											
Features	Extrapolation of global Holocene + shelf-edge (average of mass and area adjustment factors) + Halimeda mounds (mass factor)											
Timing (ka BP)	19 to 0											
Average reef thickness (m)	8–15											
Porosity (%)	35–50											
Density (kg m^−3^)	2930											
Study	Preferred area estimate Spalding et al.^56^	De Vooys^64^	Newell^73^—minimum	Spalding and Grenfell^16^	Vecsei^61^	Ryan et al^15^—minimum	Kleypas^58^—minimum	Smith^28^	Kleypas^58^—maximum	Milliman^74^	Newell^73^—maximum	Copper^65^
Reef area (km^2^)	398,095	156,995	210,261	357,445	483,601	700,872	818,618	864,876	1,045,700	2,018,510	2,102,615	2,102,615
Total volume > 130 mbsl (km^3^)	5593	2206	2954	5022	6795	9847	11,502	12,152	14,692	28,360	29,542	29,542
Net CaCO_3_ volume (km^3^)	3525	1390	1862	3165	4282	6206	7248	7658	9259	17,873	18,618	18,618
Net CaCO_3_ mass (Gt CaCO_3_)	10,328	4073	5455	9273	12,546	18,183	21,238	22,438	27,129	52,368	54,550	54,550
Net CaCO_3_ accumulation rate (Gt CaCO_3_ y^−1^)	0.6	0.2	0.3	0.5	0.7	1.1	1.2	1.3	1.6	3.1	3.2	3.2
Mass areal accumulation (kg m^−2^)	25,944	25,944	25,944	25,944	25,944	25,944	25,944	25,944	25,944	25,944	25,944	25,944

**Table 5:** Global estimates of present-day coral reef area according to different authors.

Global estimates of present-day coral-reef area	
(× 10^3^ km^2^)	
Newell^73^	150–1500
Milliman^74^	1440
Smith^28^	617
De Vooys^64^	112
Copper^65^	1500
Kleypas^58^	584–746
Spalding and Grenfell^16^	255
Ryan et al.^15^	> 500
Spalding et al.^56^	284
Vecsei^61^	345

We found that the *reef area ratio* at the shelf edge (ca. 20%) is twice the *reef area ratio* estimated for the whole GBR shelf (ca. 10% for Holocene reefs, Table 1). The structures with Holocene reefs occupy more absolute area but are sparse and separated by large extensions of flat sediment-covered submarine topography. At a global scale, previous studies suggest lower *reef area ratio* values: reef area (584 to 746 × 10^3^ km^2^) and shelf area in low latitudes (11,686 × 10^3^ km^2^) as reported in Kleypas^58^, suggesting a global *reef area ratio* of 5 to 6%. This percentage would be even lower if we apply the global reef areas of 300 × 10^3^ km^2^^61^, 255 × 10^3^ km^2^^16^ or 284 × 10^3^ km^2^^56^. The lower value of the global *reef area ratio* (5–6%) compared to the GBR values of this study (ca. 10%) could be partly explained by uncertainties in the topographic/bathymetric datasets used by Kleypas^58^ (e.g., ETOPO5^62^), and by difficulties in predicting reef habitat using the ReefHab model^58^, or by the inclusion of shelf areas that are not potential reef habitats. It is also possible that the GBR had more favourable regional conditions for reef development compared to other global locations.

The global reef area estimate of 284,000 km^2^ by Spalding et al.^56^ is lower than estimates from other authors (Table 5), but it is based on a more comprehensive dataset compared to other studies. However, this dataset originates from a collection of data from different origins and scales which brings uncertainty, especially at a local scale. Their estimates refer mainly to the area occupied by modern coral reefs and would only represent a proxy for Holocene deposits rather for than the entire *postglacial* reef system, which should include early- and mid-postglacial drowned reefs.

Applying the global reef area above and the parameters ground-truthed on the GBR shelf, we obtain a global Holocene reef deposits estimate of ca. 8100 Gt CaCO_3_ in the best-case scenario (Table 3). This is very similar to past estimates reported in Rees et al.^63^ (Table 6). However, we must also consider other calcium carbonate deposits: inter-reef carbonates, *Halimeda* bioherms and drowned reefs. Applying a similar *Halimeda*-to-reef ratio to the one estimated in the GBR (Table 1) an extra 592 Gt CaCO_3_ would be added to the global carbonate budget of the last 8 ky (Table 3). The choice of the global reef area as a parameter is critical: a simple comparison of the same calculations but using areas from other studies (Table 5) reveals a large variation in total postglacial CaCO_3_ (Holocene + drowned reefs + *Halimeda* deposits) ranging from 4073 Gt CaCO_3_^64^ to a maximum of 54,550 Gt CaCO_3_^65^ (Table 4).

**Table 6:** Global estimates of CaCO_3_ mass and/or accumulation rate of present-day shallow-water reefs, Holocene reefs and *Halimeda* bioherms according to different authors.

Study	Kinsey and Hopley^55^	Milliman and Droxler^12^	Ryan et al.^15^		Rees^13^	Hillis (^75^)	Hillis (^75^)
Feature	Holocene reefs	Holocene reefs	Holocene reefs		Holocene reefs	Present-day *Halimeda* bioherms	Total present-day *Halimeda* production
Dataset/observations	As reported in Kayanne (1992)	Productivity (here called accumulation rate) estimated for the present-day reefs	Accumulation rate and periods of significant reef growth consistent with that study		Global reef area in Spalding et al. (2001), other parameters in Rees (2006)	Global Halimeda bioherms area and production from present-day cover and accretion rates	Global Halimeda production from present-day cover and accretion rates
Timing (ka BP)	8 to 0	8 to 0	8 to 0	8 to 4	10 to 0	8 to 0	8 to 0
Reef area(km^2^)	**620,000**	**600,000**	**500,000**	**500,000**	**284,000**	**50,000**	**850,000**
Average reef thickness (m)	10	15	14	7	19	4	1
Total volume > 130 mbsl (km^3^)	6159	3826	7197	3599	5497	205	546
Porosity (%)	**50**	**50**	**50**	**50**	**50**	**50**	**50**
Net CaCO_3_ volume (km^3^)	3080	1913	3599	1799	2748	410	1092
Density (kg m^−3^)	**2890**	**2930**	**2890**	**2890**	**2900**	**2930**	**2930**
Net CaCO_3_ mass (Gt CaCO_3_)	8900	5605	10,400	5200	7970	1200	3200
Net CaCO_3_ accumulation rate (Gt CaCO_3_ y^−1^)	**0.9**	**0.7**	**1.3**	**1.3**	**0.8**	**0.2**	**0.4**
Mass areal accumulation (kg m^−2^)	14,355	9341	20,800	10,400	28,063	24,000	3765

To establish a comparison among new and past estimates, some of the values were calculated using the parameters highlighted in bold.

### Incorporating the drowned reefs in global CaCO_3_ budgets

The role of coral reefs in postglacial oceanic alkalinity changes and atmospheric CO_2_ input is poorly constrained and partly relies on CaCO_3_ deposits estimates that are uncertain. The estimates of area covered by shallow-water carbonates, from which some global estimates are derived, are mainly associated with Holocene reefs^13,16,61^ (Table 5) and ignore the more elusive (now) drowned, deeper deposits. The submerged reefs discovered in different parts of the world^21,23,24^ should be incorporated into updated postglacial CaCO_3_ deposits estimates.

In the GBR, there is strong evidence of almost continuous shallow reef accretion from ca. 30 until ca. 9.5 ka BP along the shelf edge, albeit characterised by ~ five brief demise events^21^. We estimate that the total net CaCO_3_ deposits of the submerged shelf-edge reefs are equivalent to ca. 16 to 20% of the Holocene reef deposits mass in our best-estimate scenario, and up to ca. 40% if we consider higher values of reef area ratio, postglacial reef thickness or mass areal deposits (Table 1).

If we extend the shelf edge-to-Holocene reef ratios estimated in the GBR to global scales (mass adjustment factor AF_M_ and areal adjustment factor AF_A_), we obtain a global value of 1460–1785 Gt CaCO_3_ accumulated in the drowned reefs from 19 to 10 ka BP. These results are modest compared to the combined Holocene reef deposits (ca. 8100 Gt CaCO_3_). However, given the direct evidence on large-scale pre-Holocene shelf edge reef systems in the GBR (only surveyed at scale in the last decade^30^) and past evidence of other large-scale drowned reefs in global locations^23,24^, the question of the impact of drowned reefs on the LGM-to-postglacial global CaCO_3_ budgets remains relevant.

The impact of the global reef area applied can be assessed by looking at average CaCO_**3**_ fluxes for the whole postglacial period (Table 3), which can vary from a minimum of 0.2 Gt CaCO_3_ y^−1^ (using area in De Vooys^64^) to a maximum of 3.2 Gt CaCO_3_ y^−1^ (using area in Copper^65^). However, if we split the averages between Holocene and pre-Holocene, we find the differences are of one order of magnitude (1.0 vs 0.2 Gt CaCO_3_ y^−1^; Table 3). This can be compared to some recent estimates of reef productivity of 1.9 Gt CaCO_3_ y^−1^ for the Holocene and 2.5–4.5 Gt CaCO_3_ y^−1^ for the late deglacial^19^, which were hard to reconcile with common carbon cycle models^18^. Our results are more in line with Vecsei and Berger^66^ who considered postglacial drowned reefs and reported values of 0.29–0.51 Gt CaCO_3_ y^−1^ for the Holocene and 0.15 Gt CaCO_3_ y^−1^ for the mid-late postglacial.

#### The timing of the shelf edge carbonate deposits

The timing of the CaCO_3_ deposits in the GBR, as approximated by the postglacial thickness method, suggests a possible concurrence between periods of maximum accumulation and rapid accretion rate (at ca. 12 and ca. 15.5 ka BP^21^). These were periods of high substrate availability and favourable environmental conditions for reef growth, as influenced by shelf physiography and sea-level rise^21,49^.

The postglacial thickness method cannot establish a precise chronology for CaCO_3_ mass accumulation at regional level, but it can provide a broad temporal trend. The periods of higher CaCO_3_ accumulation in the shelf edge (15.1–13.7 ka BP and 13.3–11.3 ka BP applying the 20 m maximum reef thickness assumption, Fig. 3b, Appendix 5) envelope at least two of the three episodes of increased slope of the atm-CO_2_ curve since the LGM (ca. 14.9–14.4 ka and ca. 13.0–11.5 ka BP^67^) (Fig. 3a,b). These periods of higher CaCO_3_ accumulation at the shelf edge also coincide with the episodes inferred by Ridgwell et al.^5^ in their models (17.0–13.8 ky and 12.3–11.2 ky BP). These findings are consistent with a more recent analysis of all available postglacial vertical reef accretion data (including IODP Exp. 325), which shows rapid accretion rates (9.6 to more than 20 mm yr^−1^) during these periods of higher CaCO_3_ accumulation (see Fig. S6 in Webster et al.^21^).

**Figure 6:**
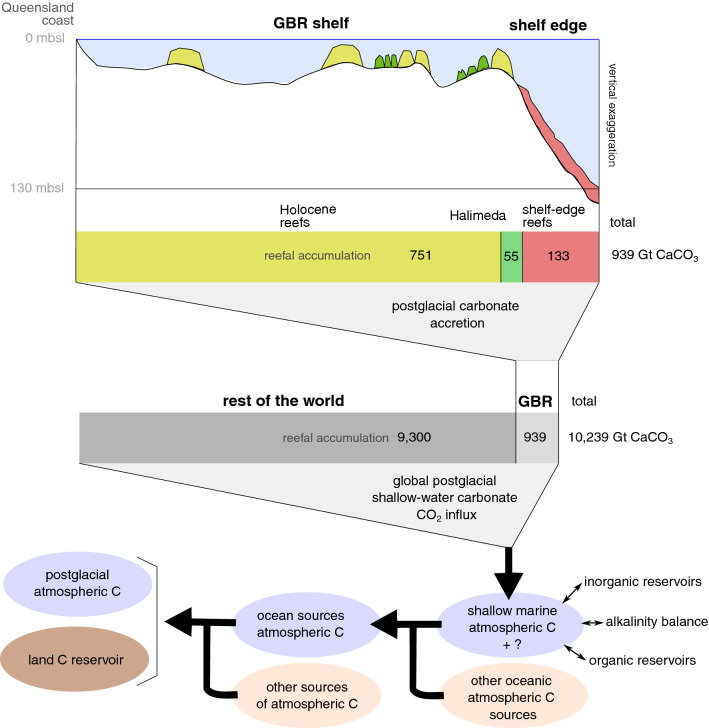
Summary of CaCO_3_ volumetrics by formation, and comparison between global and GBR regional deposits. The postglacial reefs and *Halimeda* deposits participate in the global carbon cycle by changing the alkalinity of the shallow ocean, affecting CO_2_ solubility and eventually provoking an influx of CO_2_ to the atmosphere.

Our new estimates of postglacial, pre-Holocene carbonate deposits in the GBR (ca. 130 Gt CaCO_3_) and their global extrapolations (1500 Gt CaCO_3_) suggest that pre-Holocene reef accretion is likely to be more relevant to the global CaCO_3_ budgets (hence in the global carbon cycle) than currently recognized. The impact of these deposits in the atm-CO_2_ should be assessed by global process-based carbon models that reflect the full complexity of the atmosphere–ocean–land biogeochemical cycles (Fig. 6).

## Conclusions

1. The assembled dataset provides new constraints on Holocene reef deposits in the GBR: 751 Gt CaCO_3_ as per our best estimate, varying between 520 and 1001 Gt CaCO_3_, distributed latitudinally with a strong correlation to the available shelf area.
2. The shelf-edge reefs of the GBR constitute an important portion of the postglacial shallow reef deposits: these (now) drowned reefs occupy an area of between 3000 and 12,000 km^2^, equivalent to ca. 10–45% of the total Holocene reef area in the GBR. In the GBR, these drowned reefs accumulated ca. 135 Gt of reefal CaCO_3_, equivalent to ca. 18% of the mass estimated for the more recent Holocene deposits (best estimate). The latitudinal distribution of the shelf edge reefs is also strongly correlated to shelf availability.
3. By globally extrapolating the GBR constraints, we estimate a total accumulation of 8100 Gt CaCO_3_ from Holocene reefs (best estimate), which is consistent with previously published estimates. Following from recently published results in the GBR, a minimum of ca. 590 Gt CaCO_3_ from *Halimeda* deposits should be added to the global Holocene CaCO_3_ mass, representing a 4–8% increase in the budget.
4. Global extrapolations supported by recent surveys suggest that a significant proportion of postglacial, pre-Holocene shallow-water carbonate deposits can be attributed to now drowned postglacial reefs. These deposits of ca. 1500 Gt CaCO_3_ represent 16–20% more mass in the global postglacial budget than if considering the Holocene reef deposits alone. Their inclusion in global carbon models could provide new constraints on postglacial global atmospheric and climate models.
5. Our results support a more prominent role in the postglacial carbon cycle for pre-Holocene shallow-water coral reefs. Significantly, the timing of higher CaCO_3_ deposition in the GBR is broadly coeval with two distinct episodes of postglacial atm-CO_2_ increase (ca. 14.9–14.4 ka and ca. 13.0–11.5 ka BP^67^). Any causal relationship must be confirmed by more complex, process-based models of the global carbon and other biogeochemical cycles and global surveys of drowned reefs and reef areas.

## Methods

We estimated the carbonate volume in the GBR for: (1) early postglacial reef deposits (21–10 ka); and (2) Holocene reef deposits. We also attempted a global extrapolation of these carbonate deposits based on these new and well-constrained regional GBR estimates.

Data were available across the whole GBR: bathymetry at ~ 100 m resolution^57^, GIS layers with reef locations and extensions^50^; previous GBR Holocene drill cores (see Appendix 1 for summary and Hopley et al.^68^ for data sources). We distinguished the areas at the shelf edge (defined here between the modern outer GBR reef front and the 130 m isobath) from the other areas of the shelf and extracted the corresponding bathymetric subset.

More locally, data were available for two densely surveyed control zones at the shelf edge (Noggin Passage and Hydrographers Passage; Fig. 2) where reef volumetrics and mass accumulation were estimated with a high degree of confidence for Quaternary reefs. This was possible due to: (1) availability of seismic-derived three-dimensional reconstructions of the reef patterns and volumetrics^40,47^; (2) extensive and precise chronologic database (> 580 published U-Th and ^14^C ages), lithological and petrophysical properties directly measured from the IODP Exp. 325 drill holes and cores^30,31^; (3) high-resolution (5 m) bathymetric coverage in these sites^34^; and (4) extensive knowledge on the development history and age structure of the shelf-edge reef system^21^.

For all calculations, we assumed:

- Aragonite density (ρ_A_) = 2930 kg m^−3^
- Reef formation porosity (Φ_R_) = 35%^30^.

To estimate the postglacial Pleistocene carbonate deposits, we first produced the following input for both of the estimation methods we applied:

- *Reef area ratio* [%] at the shelf-edge control zones: the percentage of reef formation area extension compared to total shelf edge area (Fig. 2). This value was obtained by digitising the area occupied by reef banks and comparing it to the total shelf-edge area. The identification of the banks was supported by the bathymetry, backscatter, seismic and GIS data^34,40,47,50^. The results of both control zones were averaged and rounded to the nearest ten to obtain a *best estimate* of 20%. To capture plausible uncertainties, arbitrary minimum and maximum values were set: a minimum *reef area ratio* of 10% equivalent to the proportion of Holocene *reef area ratio* in the whole GBR shelf; and a maximum value of twice the best estimate.
- *Formation volume* [m^3^] at the shelf-edge control zones: the volume contained between the present day, seafloor bathymetry and the antecedent basal substrate (Reflector 1) as obtained from the seismic interpretations^40,47^. The three different velocity scenarios (1700–3300 m s^−1^) to convert from seismic time to true depth^47^ were considered to obtain a minimum, best estimate, and maximum values for each set of maps.
- *Mass areal accumulation* (MAA) [kg m^−2^] at the shelf-edge control zones: these values were calculated for each control zone by transforming each *formation volume* estimate to carbonate mass (CaCO_3_ mass = *formation volume* × ρ_A_ × (1—Φ_R_)) and subsequently dividing the mass by the total shelf edge area of the control zones (MAA = mass · [shelf edge area]^−1^). The calculations were also applied for each of the main geomorphic zones^47^: inner barrier, outer barrier, terrace and shelf break combined, and inner and outer platforms combined. Three values were applied to account for plausible minimum, best and maximum scenarios: the average MAA value of all reef locations, and the minimum and the maximum MAA value in any reef location (Table 2).
- *Vertical accretion rate* [m ky^−1^] at the shelf-edge control zones: the glacial-postglacial boundary recognized in the cores and the radiometric ages measured in the Exp. 325 core samples^21^ allowed the estimation of the vertical accretion gradients (m ky^−1^) for the entire postglacial Pleistocene time period (21–10 ka) (Fig. 4, Appendix 3).
- *Maximum cumulative thickness* [m], at the shelf-edge control zones: we estimated the maximum reef thickness for the different stages of postglacial shelfedge reef development from the *vertical accretion rate* plots. For one of the estimation methods (postglacial thickness method), this became a necessary constraint to avoid unrealistic cumulative reef thickness as sea-level rise progressed (Fig. 4).
- Postglacial shelf margin *flooded area*, for each 5 m increment [km^2^]: we calculated the marine cover corresponding to past postglacial sea levels by performing surface operations in GIS software. We used the 100 m bathymetric dataset^57^ comprising the whole GBR, from northern Fraser Island to north of Cape York. We extracted the shelf-edge areas defining them as the areas between the outer GBR and the 130 m depth contour (Appendix 7). The bathymetric surface was sliced into thirty-three 50 km wide latitudinal zones after Hinestrosa et al.^49^. Each of the zones was flooded using sea levels ranging from 130 to 0 m in 5 m steps to obtain marine-flooded area in km^2^. To represent the timing of the flooded area at each sea-level increment, we applied a composite relative sea-level curve to the results in Hinestrosa et al.^49^ (Fig. 5, Appendix 6). The relative sea level was reconstructed from data in Lambeck et al.^39^, Yokoyama et al.^31^ and Webster et al.^21^ The flooded areas in each 5 m step were then matched to geological time according to sea levels represented in the relative sea-level curve (125–0 m).

The Holocene reef CaCO_3_ accumulation was calculated using the following parameters:

- *Reef area* [m^2^] from the Queensland coast to the outer GBR: the reef area was estimated from GIS layers containing polygons representing the outline of the Holocene reefs (Figs. 1 and 2, Supplementary data). This layer was obtained from a detailed interpretation of recently available satellite images and shallow bathymetry^29,50^. Continental islands and reefs belonging to the shelf edge were excluded to better approximate Holocene reef area. These features were sliced into thirty-three latitudinal zones 50 km wide to capture the latitudinal variations (Figs. 1, 3e). We assumed that the reef area polygons represent the main reef and bioclastic deposits directly related to Holocene reef growth. However, unaccounted fore- and back-reef aprons may constitute a significant portion of the reefal carbonate volume^15,51^. There are of course, uncertainties inherent to the original sources (satellite imagery and bathymetric mapping) and their interpretation, which could result in an overestimation of the reef area in the northern GBR, the under or overestimation of the extent of the bioclastic cover, or the misrepresentation of some locations as Holocene reefs when they might be older Pleistocene outcrops^50^.
- *Reef thickness* from Holocene GBR drill cores [m]: a dataset of Holocene reef thicknesses was used to estimate minimum, average and maximum thickness values for the Holocene reef thickness^68^ (Appendix 1). These drilling results show a Holocene reef veneer varying from less than 5 m to more than 25 m depending on location, with the thickest reefs recorded at around 18° S.
- *Halimeda deposits mass* [Gt CaCO_3_]: we used the most up-to-date estimate for *Halimeda* accumulations in the GBR^27^ and considered these values in our Holocene totals (Table 1).

For the global extrapolations, we also considered the following parameters:

- *Global reef area* [km^2^]: values for global reef area were extracted from the observational study by Spalding et al.^56^, but other past values (Table 5) were considered for comparison (Table 4).
- *Area adjustment factor* [%]: defined as the ratio of shelf-edge reef area to the Holocene reef area. According to our estimates in the GBR, this corresponds to 22% (Table 1).
- *Mass adjustment factor* [%]: defined as the ratio of CaCO_3_ mass at the shelf edge to the CaCO_3_ mass of the Holocene reef. According to our estimates in the GBR, this corresponds to 18% (Table 1).

### Postglacial pleistocene carbonate deposits

We followed two approaches. The postglacial-thickness method attempts to reconstruct the temporal evolution of the reef accretion by considering the change in flooded area since the LGM. The mass areal accumulation method does not consider the change in marine flooding area and outputs the cumulative postglacial volume and CaCO_3_ mass.

#### Shelf edge carbonate deposits—mass areal accumulation method

The mass areal accumulation method is based on one key assumption: that the *reef area ratio* and *mass areal accumulation* variables calculated locally at the Exp. 325 control zones are valid along the entire extension of the shelf edge.

For each latitudinal zone in the shelf edge bathymetric subset, the maximum highstand *flooded area* (FA) was multiplied by the *reef area ratio* (RAR) and *mass areal accumulation* (MAA) values to obtain CaCO_3_ accumulation values in the GBR shelf margin (Table 1).

- *Pleistocene CaCO*_*3*_* mass = FA × RAR × MAA*

#### Shelf edge carbonate deposits—postglacial-thickness method

The *postglacial-thickness method* relies on the assumption that *reef area ratio*, *vertical accretion rate* and *maximum cumulative thickness* values observed in the control zones can be extended to other locations along the GBR shelf edge. It also assumes that the parameters remain constant through time. By considering accretion rates and maximum thickness, this method allows us to approximate the temporal evolution of the shelf-edge deposits.

Firstly, the *vertical accretion rate* for each reef development episode (Webster et al., 2018) was converted to an equivalent rate relative to past sea-level steps. This allowed us to associate an incremental reef thickness to each of the 5 m sea-level steps considered. Conversion from geological age to equivalent sea level was performed using a simplified relative sea-level curve based on Lambeck et al.^39^ for ages more recent than 10 ka BP, and based on Webster et al.^21^, Yokoyama et al.^31^ for ages before 10 ka BP (Fig. 5, Appendix 6).

To obtain an estimate of reef volume at each sea-level step, we first estimated reef thickness by multiplying the converted postglacial *vertical accretion rate* (VA_SL_) by each sea-level step (5 m). Subsequently, the product of this thickness and the flooded area at each flooding stage (FA_SL_) gave us the formation volume for each one of the thirty-three latitudinal zones and for each sea-level step. The volume was scaled down by the *reef area ratio (RAR)* values (minimum, best estimate, maximum; Table 1) to obtain a measure representative of the shelf edge geomorphology as observed at the control zones. In the calculations, each flooded area had a cap on cumulative reef thickness: the m*aximum cumulative thickness* (Fig. 4). The CaCO_3_ accumulated mass for each sea-level increase was obtained by multiplying these volumes by formation net volume (1-*Φ*_*R*_) and density (*ρ*_A_):

*Pleistocene CaCO*_*3*_ *mass for each sea level (SL) between 130 and 0 m:*

- *Incremental reef volume = VA*_*SL*_ *× 5 m × FA*_*SL*_ *× RAR*
- *Incremental CaCO*_*3*_ *mass = Incremental reef volume × ρ*_A_ *× (1—Φ*_*R*_ *)*
- *Cumulative CaCO*_*3*_ *mass = Σ (Incremental CaCO*_*3*_ *mass)*

### Holocene veneer carbonate accumulation estimates

To obtain values of total *CaCO*_*3*_ mass of Holocene carbonate in the GBR, the *reef thickness* (RT) values were multiplied by the *reef area* (RA) to obtain cumulative Holocene carbonate volumes for the GBR as a whole and for each one of the thirty-three latitudinal zones (Fig. 1). Mass values were obtained by multiplying these volumes by formation net volume (1-*Φ*_*R*_) and density (*ρ*_A_) (Table 1), as summarised below:

- *Holocene reef volume = RA × RT*
- *Holocene reef CaCO*_*3*_* mass = Holocene reef volume × ρ*_*A*_* × (1 − Φ*_*R*_*)*

### Global estimates

We consider the contribution of drowned postglacial reefs in global CaCO_3_ budgets. We extrapolated the estimates and trends of CaCO_3_ deposits for the GBR to the entire globe. We used published estimates of global reef area and parameters ground-truthed by the GBR dataset. The *global reef area* (RA_GLOBAL_) was multiplied by average thickness (RT) and petrophysical parameters (ρ_A_, Φ_R_) to obtain global postglacial CaCO_3_ deposits.

We accounted for the drowned Pleistocene reefs by applying two assumptions in our calculations to obtain two equivalent results.

- Assumption 1: on average, the proportion of postglacial drowned reefs areas corresponding to a given Holocene reef area is similar across all reef provinces. We expressed this assumption as a ratio of shelf margin area to total Holocene reef area, the area adjustment factor (AF_A_),
- Assumption 2: on average, the postglacial drowned reefs mass corresponding to a given Holocene reef mass is similar across all reef provinces. We expressed this assumption as a ratio of shelf margin CaCO_3_ mass to Holocene reef CaCO_3_ mass, the mass adjustment factor (AF_M_).

The values for global reef area (*RA*_*global*_) have a large range of uncertainty as demonstrated by the range of values proposed by different authors (Table 5). We consider those by Spalding et al.^56^ more accurate given the ground-truthing datasets. The factors that we applied on the global area (*AF*_*A*_*, AF*_*M*_) have a large associated uncertainty: despite the evidence for drowned reefs in other geographical locations, the exact global extension and morphology of drowned reefs is not well constrained. Other carbonate provinces might differ in morphology, in accretion trends and in the proportion of Pleistocene reefs present along their margins compared to the more recent Holocene deposits of those provinces.

- *Global postglacial CaCO*_*3*_* accumulation = RA*_*global*_* × RT*_*GBR*_* × ρ*_*A*_* × (1—Φ*_*R*_*)*
- *Assumption 1:*
  - *Area-adjusted global postglacial CaCO*_*3*_* mass = RA*_*global*_* × RT*_*GBR*_* × *ρ_A_* × (1—Φ*_*R*_*) × (1 + AF*_*A*_*)*
- *Assumption 2:*
  - *Mass-adjusted global postglacial CaCO*_*3*_* mass = RA*_*global*_* × RT*_*GBR*_* × ρ*_A_* × (1—Φ*_*R*_*) × (1 + AF*_*M*_*)*.

### On CO_2_ and total C estimates

The chemical equilibrium of the shallow ocean is complex, with the concentration of the main inorganic carbon species (CO_2_, HCO_3_^−^, CO_3_^−^) varying according to temperature, salinity and pressure^69,70^.

According to the coral reef hypothesis^6,71^, reefal CaCO_3_ accretion provides CO_2_ to the environment by increasing the concentration of CO_2_ in the ocean water. The equivalent CO_2_ and C mass based on the stoichiometry of the chemical reaction: Ca^2+^  + 2HCO_3_^−^ = => CaCO_3_ + CO_2_ + H_2_O^6,66^ gives an incomplete picture because the exact proportion would depend on physical conditions that would have varied during the postglacial period. Experimentally, it has been demonstrated that for each mole of CaCO_3_ precipitated in seawater only a fraction of a mole of CO_2_ is fed to the surrounding waters due to the buffering effect of marine water^8,72^. It is then unclear how much of this carbon is released into the atmosphere, especially at centennial timescales^66,72^. The pathways for the released CO_2_ molecules are varied—they can be absorbed into inorganic or organic marine carbon cycles, or they can also be transferred into the atmosphere by the balancing of the partial pressure of CO_2_. We have not attempted to quantify the corresponding postglacial CO_2_ contribution to the surrounding waters and to the atmosphere. This would require more complex carbon models which are beyond the scope of this study.

## Supplementary Information

Supplementary Information.

Supplementary Information 1.

Supplementary Information 2.

Supplementary Information 3.

Supplementary Information 4.

Supplementary Information 5.

Supplementary Information 6.

Supplementary Information 7.

Supplementary Information 8.
