# Author Correction: New constraints on the postglacial shallow-water carbonate accumulation in the Great Barrier Reef

**DOI:** 10.1038/s41598-022-06337-x

**Published:** 2022-02-01

**Authors:** Gustavo Hinestrosa, Jody M. Webster, Robin J. Beaman

**Affiliations:** 1 Geocoastal Research Group, School of Geosciences, The University of Sydney, Sydney, Australia; 2 College of Science and Engineering, James Cook University, Cairns, Australia

Correction to: *Scientific Reports* https://doi.org/10.1038/s41598-021-04586-w, published online 18 January 2022

The Supplementary Information 3 file published with this Article contained an error where irrelevant data samples, incorrect or out-of-date references, and inconsistent notations obscured the results of the postglacial accretion trends and affected their reproducibility. The original Supplementary Information 3 file is provided below.

This error has now been corrected in the Supplementary Information 3 file that accompanies the original Article.

## Supplementary Information

Supplementary Information 3.

