# Supplementary material for: New constraints on the postglacial shallow-water carbonate accumulation in the Great Barrier Reef
Source: Sci Rep. 2022 Jan 18;12:924. doi: 10.1038/s41598-021-04586-w (PMC8766595; doi:10.1038/s41598-021-04586-w)
Supplement: Supplementary file 1 — Supplementary Information. [file 41598_2021_4586_MOESM1_ESM.docx]

Supplementary material

- Appendix 1: Holocene thickness from GBR core data
  - File: *appendix1_cores_holocene_thickness_GBR.xlsx*
- Appendix 2: Holocene CaCO_3_ volumetrics and mass
  - File: *appendix2_holocene_volumetrics.xlsx*
- Appendix 3: Postglacial accretion trends and thickness from core data
  - File: *appendix2_postglacial_thickness_trends.xlsx*
- Appendix 4: Postglacial CaCO_3_ estimates using past published global reef areas
  - File: *appendix3_global_volumetrics_calculations_different_areas.xlsx*
- Appendix 5: Shelf edge CaCO_3_ volumetrics and mass calculations
  - File: *appendix4_shelf_edge_carbonate_calculations.xlsx*
- Appendix 6: Composite sea level curve
  - File: appendix5_sea_level_curves_Hinestrosa_et_al_2021.xlsx
- Appendix 7: GIS datasets
  - Polygons highlighting the shelf edge area as per definition
  - Reef and reef wedge polygons as per detailed interpretation of recently available satellite images and shallow bathymetry [^29^](#_ENREF_29)^,^[^50^](#_ENREF_50).
