# Supplementary material for: New constraints on the postglacial shallow-water carbonate accumulation in the Great Barrier Reef
Source: Sci Rep. 2022 Jan 18;12:924. doi: 10.1038/s41598-021-04586-w (PMC8766595; doi:10.1038/s41598-021-04586-w)
Supplement: Supplementary file 9 — Supplementary Information 8. [file 41598_2021_4586_MOESM9_ESM.docx]

# Appendix 1

“*New constraints on the shallow-water postglacial CaCO_3_ accumulation in the Great Barrier Reef”*

Gustavo Hinestrosa, Jody M. Webster and Robin J. Beaman

Braithwaite, C. J., Dalmasso, H., Gilmour, M. A., Harkness, D. D., Henderson, G. M., Kay, R. L. F., Kroon, D., Montaggioni, L. F. and Wilson, P. A., 2004. The Great Barrier Reef: the chronological record from a new borehole. Journal of Sedimentary Research, 74(2), pp.298-310.

Davies, P. J. and Hopley, D., 1983. Growth fabrics and growth rates of Holocene reefs in the Great Barrier Reef.: BMR Journal of Australian Geology and Geophysics, v. 8, p. 237-251.

Davies P. J., Marshall J. F., Radke B, 1973. Growth rate: reefs of the central and northern province. Proceedings Inaugral Great Barrier Reef Conference, Townsville. JCU Press, Townsville, Australia, pp 95–98.

Davies P. J., Marshall JF and Hopley D, 1985. Relationships between reef growth and sea level in the Great Barrier Reef: 5th International Coral Reef Congress, v. 3, p. 95-103.

Graham, T. L., 1993. Geomorphological Response of continental shelf and coastal environments to the Holocene transgression—Central Great Barrier Reef. Ph.D. thesis, James Cook University, Australia, p 206.

Harris, P. T., Davies, P. J., and Marshall, J. F., 1990. Late Quaternary sedimentation on the Great Barrier Reef continental shelf and slope east Townsville, Australia: Marine Geology, v. 94, p. 55-77.

Harvey, N., and Hopley, D., 1981. The relationship between modern reef morphology and a pre-holocene substrate in the Great Barrier Reef: Proceedings of the Fourth International Coral Reef Symposium, Manila, v. 1, p. 550-554.

Hopley, D., 1983. Preliminary results of four year drilling programme on the Great Barrier Reef, *in* Proceedings of the Inaugural Great Barrier Reef Conference, 1983, Editor: [Joseph T. Baker](https://www.google.co.uk/search?safe=active&hl=en&sxsrf=ALeKk00CLmTeXEFZwXUCi3FneC5oYkTGcQ:1618174594161&q=Joseph+T.+Baker&stick=H4sIAAAAAAAAAONgVuLRT9c3NDSujC83tSxexMrvlV-cWpChEKKn4JSYnVoEAAz79EEiAAAA&sa=X&ved=2ahUKEwiHiOuOivfvAhWJ4IUKHe9FAe4QmxMoADAOegQICxAC).

Hopley, D., and Barnes, R., Structure and development of a windward fringing reef, Orpheus Island, Palm Group, Great Barrier Reef, *in* Proceedings Proceeding of the Fifth Coral Reef Congress, 1985, Volume 3, p. 141-146.

Hopley, D., and Harvey, N., Radiocarbon ages and morphology of reefs tops in the Great Barrier between 14˚39 S and 20˚45 S: Indicators of shelf neotectonics?, *in* Proceedings of the Fourth International Coral Reef Symposium, Manila, 1981, Volume 1, p. 523-530.

Hopley, A. M. Slocombe, F. Muir & Grant, C., 1983. Nearshore fringing reefs in north Queensland, Coral Reefs, volume 1, pages 151–160.

Hopley, D., Muir, F. J., and Grant, C. R., 1984. Pleistocene foundations and Holocene growth of Redbill, South Central Great Barrier Reef: Search, v. 15, no. 9-10.

Hopley, D., Smithers, S. G. and Parnell, K., 2007. The geomorphology of the Great Barrier Reef: development, diversity and change. Cambridge University Press.

Johnson, D. P., and Risk, M. J., 1987. Fringing reef growth on a terrigenous mud foundation, Fantome Island, central Great Barrie Reef, Australia: Sedimentology, v. 34, p. 275-287.

Johnson, D., Cuff, C., and Rhodes, E., 1984. Holocene reef sequences and geochemistry, Britmart Reef, central Great Barrier Reef, Australia: Sedimentology, v. 31, p. 515-529.

Kleypas, J. A., Hopley, D., Reef development across a broad continental shelf, southern Great Barrier Reef, Australia, *in* Proceedings Proceedings of the 7th Inter. Coral Reef Symposium, 1993, Volume 2, p. 1129-1141.

Marshall, J., and Davies, P. J., 1982. Internal structure and Holocene evolution of One Tree Reef, Southern Great Barrier Reef: Coral Reefs, v. 1, p. 21-28.

Partain, B. R., and Hopley, D., 1989. Morphology and development of the Cape Tribulation Fringing Reefs, Great Barrier Reef: Australia Great Barrier Reef Marine Park Authority, Technical Memorandum, 21.

Smithers, S., and Larcombe, P., 2003. Late Holocene initiation and growth of a nearshore turbid-zone coral reef: Paluma Shoals, central Great Barrier Reef, Australia: Coral Reefs, v. 22, no. 4, p. 499.

Woodroffe, C.D., Kennedy, D.M., Hopley, D., Rasmussen, C.E. and Smithers, S.G., 2000. Holocene reef growth in Torres Strait. Marine Geology, 170(3-4), pp.331-346.
